# Using Digital Health Technologies to Monitor Pain, Medication Adherence and Physical Activity in Young People with Juvenile Idiopathic Arthritis: A Feasibility Study

**DOI:** 10.3390/healthcare12030392

**Published:** 2024-02-02

**Authors:** Sonia Butler, Dean Sculley, Derek Santos, Xavier Girones, Davinder Singh-Grewal, Andrea Coda

**Affiliations:** 1 School of Bioscience and Pharmacy, University of Newcastle, Ourimbah, NSW 2258, Australia; dean.sculley@newcastle.edu.au; 2 School of Health Sciences, Queen Margaret University, Edinburgh EH21 6UU, UK; dsantos@qmu.ac.uk; 3 Department of Research, Universities de Catalunya, Generalitat de Catalunya, 08003 Barcelona, Spain; xaviergirones@gencat.cat; 4 Department of Rheumatology, Sydney Children’s Hospitals Network (Randwick), Randwick, NSW 2031, Australia; davinder.singhgrewal@health.nsw.gov.au; 5 Department of Rheumatology, Sydney Children’s Hospitals Network (Westmead), Westmead, NSW 2145, Australia; 6 John Hunter Children’s Hospital, New Lambton Heights, NSW 2305, Australia; 7 Discipline of Child and Adolescent Health, University of Sydney, Camperdown, NSW 2050, Australia; 8 School of Women’s and Children’s Health, University of NSW, Sydney, NSW 2052, Australia; 9 School of Health Sciences, University of Newcastle, Callaghan, NSW 2308, Australia; andrea.coda@newcastle.edu.au; 10 Equity in Health and Wellbeing Research Program, The Hunter Medical Research Institute (HMRI), Newcastle, NSW 2305, Australia

**Keywords:** digital health, juvenile idiopathic arthritis, paediatric, pain, medication adherence, physical activity, app, smartwatch

## Abstract

Juvenile idiopathic arthritis can be influenced by pain, medication adherence, and physical activity. A new digital health intervention, InteractiveClinics, aims to monitor these modifiable risk factors. Twelve children, aged 10 to 18 years, received daily notifications on a smartwatch to record their pain levels and take their medications, using a customised mobile app synchronised to a secure web-based platform. Daily physical activity levels were automatically recorded by wearing a smartwatch. Using a quantitative descriptive research design, feasibility and user adoption were evaluated. The web-based data revealed the following: Pain: mean app usage: 68% (SD 30, range: 28.6% to 100%); pain score: 2.9 out of 10 (SD 1.8, range: 0.3 to 6.2 out of 10). Medication adherence: mean app usage: 20.7% (SD, range: 0% to 71.4%), recording 39% (71/182) of the expected daily and 37.5% (3/8) of the weekly medications. Pro-re-nata (PRN) medication monitoring: 33.3% (4/12), one to six additional medications (mean 3.5, SD 2.4) for 2–6 days. Physical activity: watch wearing behaviour: 69.7% (439/630), recording low levels of moderate-to-vigorous physical activity (mean: 11.8, SD: 13.5 min, range: 0–47 min). To conclude, remote monitoring of real-time data is feasible. However, further research is needed to increase adoption rates among children.

## 1. Introduction

Juvenile idiopathic arthritis (JIA) is the most common paediatric rheumatological disorder, impacting the lives of 6000 Australian children [1]. JIA is classified as an arthritis of unknown origin, manifesting before the age of 18 years and lasting for more than a period of six weeks [2,3]. Diagnosis is concluded after all other possible causes are eliminated [4].

JIA is not the same as adult arthritis [5]. JIA comprises a group of heterogeneous disorders with seven subtypes. Each subtype has different phenotypes, symptoms, and trajectories [5,6]. Commonly, children experience pain, stiffness, and fatigue [7], which can impact their participation in daily activities and school [8,9], whilst an active disease can cause serious joint destruction, malformation of the bones, and changes in the child’s growth patterns, impairing their functional ability [6,10,11,12]. Unfortunately, at present, there is no definitive cure. Though, over the last decade, due to a better understanding of inflammatory arthritides and cytokine networks, the medications available to promote clinical remission have greatly improved, gaining, for many, good clinical outcomes [4,11,12]. Nonetheless, 50% of children are still reaching adulthood with active disease [1,11,13], and 10–30% gain a moderate or severe functional disability [6,14]. Achieving a good patient outcome is dependent upon good self-management behaviour [14] and can be compromised by the following modifiable factors: pain, medication adherence, and reduced levels of physical activity [14,15,16]. Recent advances in digital health have the potential to improve patient monitoring, treatment adherence, and positive health behaviours [17,18,19], which are the main objectives of paediatric rheumatology disease management.

### 1.1. Utilising Digital Health Technology

Wearable devices, such as smartwatches, are now capable of monitoring and assessing in real time a child’s crucial health indicators. They are aesthetically pleasing and can gather disease-specific information discreetly in day-to-day life [20]. In fact, in most consumer-grade smartwatches, there are applications and inbuilt sensors that can collect a wide array of measurable physiological, behavioural, and environmental information [21]. This rich corpus of data can then be processed actively or reactively. Active, meaning the data can be sent continuously by Bluetooth, from sensors in the smartwatch to sensors in a smartphone, where the information can be collaborated and stored [20]. This allows the user to view this information as a daily, weekly, or monthly summary and has the potential to enable the user to gain a better understanding of their disease and self-management behaviour [20,22].

Secondly, this information stored on the smartphone can be automatically uploaded to a secured online health platform and used as a clinical decision-making tool [20,23,24]. The platform can enable the information to be viewed by the healthcare team, or the platform’s programming interface can create a reactive response. This means the user receives a response according to a pre-established algorithm. This response could be an SMS to the smartphone and/or smartwatch, alerting the user, or a phone call to the user, caregiver, doctor, or hospital [20]. The benefits of this active and reactive information are that it is not restricted by time, location, or situation [20]. This portability and connectivity make smartwatches a powerful new tool [25] that could be used to redefine chronic disease management, and access to this technology is already readily available [22]. Market research reports that 22.5% (range: 5.9% to 33.4%) of people globally now own a smartwatch [26], with a predictive forecast that this number will increase substantially over the next few years [27].

However, only a small number of studies have begun to explore the potential of smartwatches, and most are limited in their rigor. A recent systematic review, for example, noted that most studies were conducted on healthy participants (71%, 12/17) in a laboratory or hospital setting (65%, 11/17), rather than by those living with targeted health conditions in the real-world home setting [21]. Most studies also did not focus solely on children (12%, 2/17, and 15%, 3/20) [21,28], suggesting the need for more customised applications that are tailored towards specific health problems and children.

### 1.2. InteractiveClinics—A Web-Based Platform for Digital Health Research

InteractiveClinics is a newly developed digital health web-based platform that aims to support international digital health research and enable data communication. InteractiveClinics was developed by academics from the University of Newcastle, Australia, and the University of Manresa (Catalonia), Spain, with paid support from BitGenoma Ltd. Digital Solutions (Barcelona, Spain) for software engineering. InteractiveClinics can support app development for both Apple and Android operating systems (OS). The cost of development for the platform, app, and important server protection was approximately AUD 75,000.

For JIA, InteractiveClinics was utilised with the intention to support chronic disease management by improving communication between the patient and the healthcare team by connecting a commercially available smartwatch to a customized app to prompt and/or monitor the following three modifiable risk factors associated with poor health outcomes: pain, medication adherence, and physical activity. To the best of our knowledge, this is the first study that aims to provide a multimodal approach to support the management of JIA. Therefore, the aim of this study is to evaluate InteractiveClinics feasibility.

### 1.3. Definition

The World Health Organization defines digital health feasibility testing as follows:

(1.) Assessing if the intervention works as intended and is error-free;
(2.) Demonstrating end-user acceptance [29].

## 2. Materials and Methods

### 2.1. Study Design

To ensure the success of this digital health intervention, the WHO’s step-up approach was employed to aid prototype development and improve the quality of the intervention [29]. Typically, testing starts at a very early stage in the intervention’s life cycle, and these steps include the following: monitoring functionality and stability, then evaluating feasibility, usability, efficacy, and effectiveness [29]. This study focused on the feasibility stage of testing, using a quantitative descriptive design to analyse the data collected on the InteractiveClinics web-based platform, identify any adjustments in the system needed, affirm the consistency and data integrity, and support future deployment of the intervention [29].

### 2.2. Sampling

Convenience sampling was used to recruit 12 participants to meet the WHO’s recommended sample size (≥10 participants) for digital health feasibility studies [29]. No power calculations were conducted because this early stage of testing only focused on quality improvement. The inclusion criteria included a diagnosis of JIA according to the International League of Associations for Rheumatology (ILAR), an age range from 10 to 18 years, and good comprehension of the English language. The exclusion criteria included a cognitive or physical impairment that would affect the participants’ ability to use digital technology or a severe visual impairment (for example, uveitis). The recruitment criteria did not consider the child’s current level of pain or physical activity.

### 2.3. Intervention

#### 2.3.1. InteractiveClinics Setup

To participate in the study, all participants were provided with the following materials:

1. Apple watch (series 3), selected because it had a water-resistance rating of 50 metres and did not need to be removed for low-intensity activities such as swimming [30]. An activity that is safe and effective for children with JIA [31].
2. Refurbished Apple iPhone (SE, 2016), loaded with pre-paid credit (AUD 30), selected because of the purchase price, a 4-inch screen, and the simplicity of the home button for children.
3. Interactive Clinics app, free to download from the Apple or Google Play Store (already pre-set on the iPhone).
4. Personal password, to access the secure, password-locked, web-based platform and to the summary of the data collected from the app.
5. Training and support (approximately 10 min), which included demonstrations and written instructions. The contact details of SB were also placed within the phone’s address book, to gain further support if needed.
6. Prepaid envelope, to return the watch and phone at the end of the study.

#### 2.3.2. InteractiveClinics Modules

**Figure 1:**
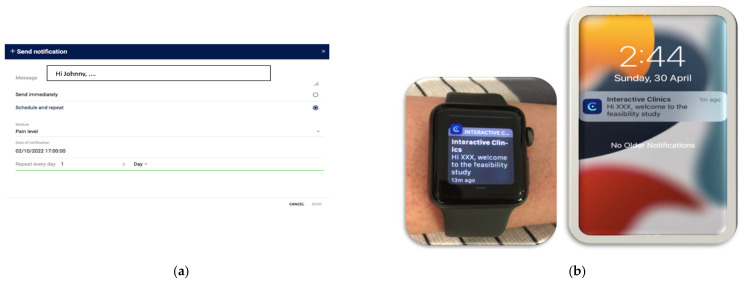
InteractiveClinics personalised automatic notification system. (**a**) Setting up personalised notifications on the web-based platform. (**b**) Notifications sent to the smartwatch and phone.

InteractiveClinics aims to motivate children to record their pain, take their medication, and increase their participation in physical activity. InteractiveClinics presents these three key areas as three modules: pain level, medication adherence, and physical activity, within the phone app and on the web-based platform. To promote daily adoption of the intervention, personalised notifications were sent to the participants at the time that they requested (Figure 1).

#### 2.3.3. Pain Level Module

**Figure 2:**
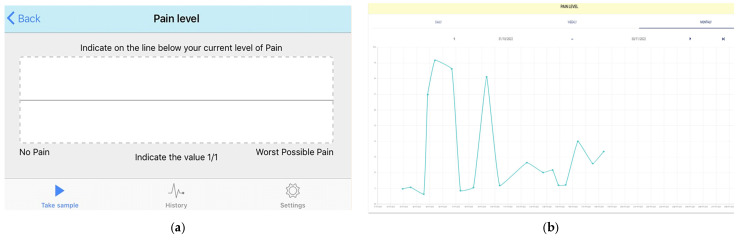
Pain level module. (**a**) Participant recording their pain level on the *eVas*. (**b**) An example of pain monitoring on the web-based platform.

- Set up: Participants self-selected their pain notification time, and this was entered by the researchers (SB) into the web-based platform. A personal message to each participant was also created.
- During the study period: Personalised notifications were sent daily to the smartwatch and phone to remind participants to record their pain.
- User actions: Pain levels were recorded in the app using the validated electronic visual analogue scale (*eVas*) [32,33,34]. *eVas* utilises a simple horizontal line with defined pain limits. The left endpoint indicates no pain, and the right endpoint indicates the worst possible pain. This reporting scale has been found to be highly reliable and consistent with the original paper-based visual analogue scale (VAS) [32,34] (Figure 2).
- Data feedback: Visual feedback was then provided to the participants, displaying their daily pain level using the numerical score (0–10) and a monthly pain graph. The same information was available on the web-based platform, enabling the parent/carer and paediatric PR research teams to longitudinally monitor pain trends (Figure 2) (Also see Table 1 below).

#### 2.3.4. Medication Adherence Module

**Figure 3:**
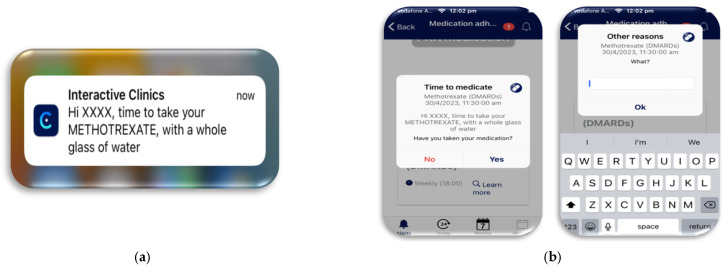
Medication adherence module. (**a**) Personalised medication reminder. (**b**) If the participant clicked no, they did not take their medication, a free text box opened to allow participants to record their reasons, for example, side effects.

- Setup: Each participant’s medication and administration times were entered into the web-based platform. This information could be viewed by the participants through the app.
- During the study: Personalised notifications were sent to the watch and phone to remind the participants that their medications were due and how the medication should be taken. For example, methotrexate, with a whole glass of water (Figure 3).
- User actions: The participants were instructed to record their medication administration in the app. If the participants did not take their medication, they were then asked to provide a reason why, either from a pre-programmed list or a free text box to allow a unique answer (Figure 3). The medication module also allowed for additional pro-re-nata (PRN) medications to be recorded in case breakthrough pain medications were needed.
- Data feedback: Visual feedback was then provided to the participants, displaying their daily, weekly, and monthly medication usage in the app. Through the web-based platform, the parent/carer and PR research teams could also review the same information, allowing them to graphically track medication adherence (Figure 3) (Also see Table 1 below).

#### 2.3.5. Physical Activity Level Module

**Figure 4:**
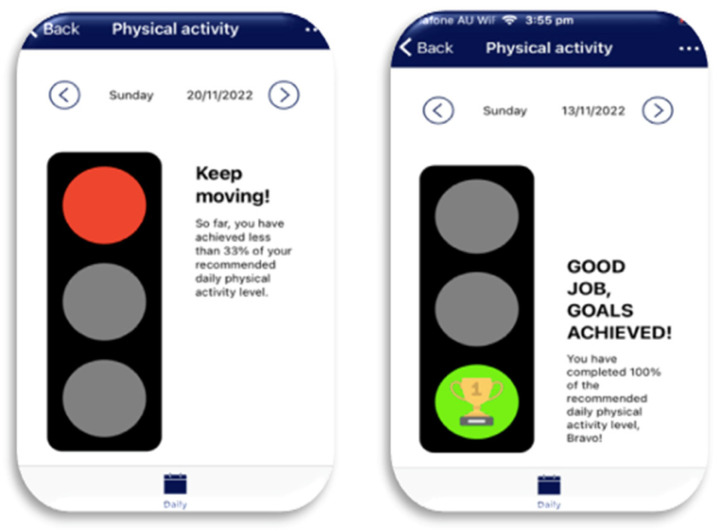
Physical activity module within the app.

- Setup: Each participant’s personal physical activity goals were set during the training session in accordance with their age and Australia’s Physical Activity and Sedentary Behaviour Guidelines [35]. Corresponding pamphlets were also provided to supply a variety of achievable exercise options, such as walking to school, and education on sedentary behaviour, such as extended sitting [35].
- User actions: Through participants simply wearing the watch each day, physical activity levels were automatically recorded by the biosensors in the watch measuring heart rate and the three-axis accelerometer measuring changes in velocity [36]. These data were then transferred from the phone’s commercial fitness app to the InteractiveClinics app and web-based platform. This synchronisation was dependent on a once-only authorisation on the phone and internet connectivity.
- Data feedback: Visual feedback was provided to participants in the app to motivate the participants to reach their daily goals (Figure 4). The web-based platform was more detailed by graphically displaying the participant’s daily and monthly physical activity levels, making these data available to the participant, parent/carer, and the research team (Also see Table 1 below).

**Table 1:** Overview of the data collection method.

Module	Persuasive Influence	Data Collection Method
Pain level	Customised notifications to watch and phone	User action via app
Medication adherence	Customised notifications to watch and phone	User action via app
Physical activity levels	User action—wearing the watch	Automatic

### 2.4. Participant Recruitment

First, participants were recruited from a monthly Paediatric Rheumatology outpatient’s clinic in a regional children’s hospital, between July and November 2022. Then, because of the cyclical return of the same children to the clinic, the final week of recruitment moved to a tertiary children’s hospital in a major capital city. Both clinics were in NSW, Australia.

Of the potential 15 participants meeting the inclusion criteria, 12 participants, aged 10 to 18 years (mean: 14.2, SD: 3.1, female: 66.7%, 8/12), agreed to participate and completed the study. JIA subtypes included the following: polyarthritis (Rheumatoid Factor (Rh − ve)) (41.7%, 5/12), oligoarthritis (33.3%, 4/12), enthesitis-related (8.3%, 1/12), polyarthritis (Rh + ve) (8.3%, 1/12), and psoriatic (8.3%, 1%). The disease duration ranged from 5 months to 10 years (mean 4.9 years).

Most participants (91.7%, 11/12) were prescribed regular medications for pain (58.3%, 7/12) and disease activity (50%, 6/12). The frequency of medication administration varied from once a day (75%, 9/12), to twice a day (8.3%, 1/12), every second day (8.3%, 1/12), once a week (33.3%, 4/12), twice a week (8.3%, 1/12), or PRN, when needed (66.7%, 8/12).

### 2.5. Follow-Up of Participants

Participation in the study involved wearing a smartwatch for 2 to 4 weeks. This time period varied due to time restraints around the COVID-19 pandemic and limitations on the loan equipment (smartwatch and phone).

Initially, the baseline demographic data were collected at recruitment (T_0_). This included the following: date of birth, gender, JIA subtype, disease duration, prescribed medications, and administration times. Then, follow-up for all participants took place across the study period on day one (T_1_), day two (T_1_), the second-last day of the study (T_3_), and the last day of the study (T_3_), depending on whether it was the 2- or 4-week study period.

T_1_—A notification was sent to the participants, welcoming them to the first day of the study. SB viewed the participants pain, medication adherence, and physical activity levels entries on the web-based platform. If SB noticed any missed entries, a text message was sent, offering the participants further training or technical support. SB also asked how they would like this support to be delivered, by text message, phone call, or email.

T_2_—SB again viewed the participants’ pain, medication adherence, and physical activity levels entries on the web-based platform, and if not completed, it offered support by text message.

T_3_—A notification was sent explaining that the study will conclude tomorrow evening.

T_4_—A notification was sent, thanking participants for their contribution to the study and reminding participants to return the smartwatch and phone to SB in the prepaid envelope provided at recruitment.

In future studies, notifications/messages sent at T_1_, T_3_, and T_4_ could be pre-set as notifications at T_0_.

### 2.6. Ethical Considerations

Ethics approval was granted by the Hunter New England Research Ethics Committee (ref no: ETH01035) to conduct this study at two children’s hospitals in NSW, Australia. All participants and/or their parent, signed an informed consent. To ensure anonymity, all identifying details were removed and replaced with a participant number. Participants were able to withdraw from the study at any time, without prejudice, by simply returning the watch and phone in the supplied, pre-paid envelope.

### 2.7. Outcomes

#### 2.7.1. Primary Feasibility Outcomes

(1.) App usage: Measured by the percentage of patients who used InteractiveClinics daily. Only missed medication administration entries were recorded by the system.
(2.) Confirmation of data integrity: Measured by the percentage of missing or incomplete data between the web-based platform and watch and phone’s Apple health app.
(3.) Preliminary interpretation: By presenting the initial results of the study population’s data collected on InteractiveClinics web-based platform.
  - For the pain module, using *eVAS* scores.
  - For the medication module, the rate of medication administration.
  - For the physical activity level module, daily kilojoules (light physical activity), moderate-to-vigorous physical activity, and stand hours.
(4.) Watch wearing behaviour: Measured by the number of days that the watch was worn across the study period.

#### 2.7.2. Secondary Outcomes

(1.) Technical errors: measured by the percentage of participants’ needing technical support after intervention training.

### 2.8. Data Collection

A data extraction Excel (Microsoft) form was designed to collect the relevant data from the web-based platform. The platform was able to illustrate the exact day and time of pain levels and medication administration responses that were entered into the app. The platform could also identify the days when no input was provided. The daily physical activity levels were also dated but not time-stamped due to the continuous and automatic uptake of data throughout the day.

Data extracted included the following: pain eVas scores; medication administration recordings; reasons for non-adherence; PRN medications; daily physical activity levels, including kilojoules, light physical activity levels; moderate-to-vigorous physical activity levels; and hourly stand goals. Data were also extracted from the research journal to identify the errors reported by the participants. Data extraction was completed by SB and checked by the research team.

Table 2 outlines the schedule of data collection across the study period.

### 2.9. Data Analysis

Descriptive statistics were used to summarise and report the data collected on the web-based platform. App usage rates were determined by examining the number of observed entries against the expected number of entries. Data integrity was calculated by comparing the results in Apple’s health kit to the final results on the web-based platform to identify differences and the portion of missing data. The preliminary interpretation of the continuous pain and physical activity data was summarised by measuring the central tendency to determine the mean, standard deviation, and range. Medication adherence rates were determined by calculating the number of doses administered against the number of doses prescribed. The watch wearing behaviour was calculated by the number of days that the watch was worn across the study period. Technical issues were identified by the ‘number of participants raising problems’ divided by ‘the total number of participants’ × 100 to form a percentage.

In addition, all of the technical problems reported were recorded in a research journal. Some participants also provided written feedback when they returned the watch, explaining their watch wearing behaviour. This feedback is presented as raw qualitative excerpts due to the limited amount of data [37].

## 3. Results

### 3.1. Pain Level Module

App usage: The mean app usage for the pain module was 66.8% (SD: 30, range from 28.6% to 100%).

Confirmation of data integrity: There were also no errors between the pain score recorded using *eVAS* in the app and the pain scores recorded on the web-based platform.

Preliminary interpretation: Across the twelve participants, a mild mean pain score of 2.9 out of 10 (SD: 1.8, range from 0.3 to 6.2 out of 10) was recorded on the web-based platform.

### 3.2. Medication Adherence Module

App usage: The mean app usage for the medication module was 20.7% (SD: 26, range from 0% to 71.4%). Three participants (25%, 3/12) failed to use the medication module properly, and another three (25%, 3/12) needed to re-connect the app to the web-based platform. However, these participants were unable to backdate their responses.

Confirmation of data integrity: There were no errors recorded between medication entries in the app and on the web-based platform.

#### Preliminary Interpretation

- Daily medication adherence: Overall, the web-based platform recorded 39% (71/182) of the expected medication entries.
- Twice-a-day medication adherence: One participant (8.3%, 1/12) required the same medication twice a day. This medication frequency could not be entered into the system due to a coding fault.
- Weekly medication adherence: Three medication entries of the expected eight (37.5%, 3/8) were recorded by three participants.
- Reasons for non-adherence: Only one participant utilised the text box to provide their reason for non-adherence, explaining they ‘arrived home late’ (Participant 6).
- PRN medication monitoring: Eight participants in this study often needed PRN medications for their pain or stiffness. Over 14 days, the web-based platform recorded four participants needing additional medication (mean: 3.5, SD: 2.4), for 2 to 6 days. When PRN medications were not needed, ten responses were also seen, for example, (there was) “no need” (Participant 7).

### 3.3. Physical Activity Module

App usage: Overall, 435 daily physical activity entries were observed on the web-based platform, accounting for 69.7% (439/630) of the possible 630 entries.

Confirmation of data integrity: There was a reporting error of 10% (63/630); 63 of these entries did not match the data collected from the smartwatch; 27 were an underestimation of daily physical activity levels; and 36 were missing the recorded data. This was due to the app not running as a background app and needing internet connectivity.

Preliminary interpretation: These entries included kilojoules (kJ) due to light physical activity (mean: 244, SD: 104.8 kJ, range: 86–415 kJ) per day, exercise (moderate-to-vigorous physical activity, mean: 11.8, SD: 13.5 min; range: 0–47 min) per day and hourly stand goals (mean: 8.8, SD: 3.6 h; range: 1–13 h) per day to inhibit sedentary activity.

### 3.4. Smartwatch Wearing Behaviour

Smartwatch wearing behaviour averaged at 13 days (ranging from 4 to 26 days) over the 14- to 28-day study period. Initially, the study period was set to 4 weeks (28 days), and three participants were included. The watch wearing behaviour for two participants was 92.9% (26/28 days) and for one participant, 64.3% (18/28 days). One participant continued to wear the watch for 5 days after the conclusion of the study. These data collected after the study period were not included in the data analysis.

**Figure 5:**
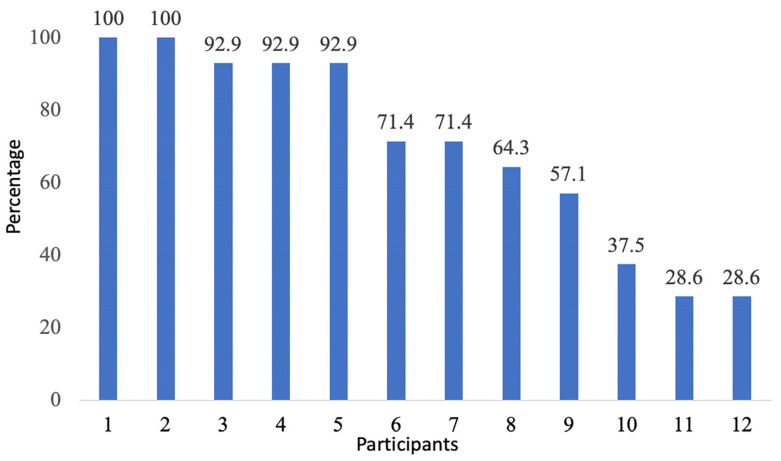
Participants watch wearing behaviour across the study period.

Then, the study period was reduced to 2 weeks (14 days) for the other nine participants because of the COVID-19 pandemic restrictions. Over this time period, the two youngest participants (aged 10 years) wore the watch every day (100%, 14/14 days). Another participant missed 1 day (92.9%, 13/14), two participants missed 4 days (71.4%, 10/14) and one missed 6 days (57.1%, 8/14 days) (Figure 5). The main reason was because they “forgot to wear [the watch]”. The three oldest female participants had the lowest rate of watch wearing behaviour, ranging from 28.6% (4/14) to 35.7% (5/14). They stopped wearing the watch after 4 to 5 days, missing 9 to 10 days because they “did not like the feeling of a watch” on their arm, and it was “too big” and kept “flipping around”. Four participants continued to wear the watch beyond the 2-week study period: two participants for 1 day, another two for 3 days. These data collected beyond the study were not included in the data analysis.

### 3.5. Ongoing Technical Support

Over the first week of the study, all participants (100%, 12/12) required additional technical support to use InteractiveClinics. For most participants, the preferred method of contact was text messages (75%, 8/12). Only the youngest participants in the study (aged 10 to 11 years) gained this support via their parents, through either text message (16.7%, 2/12), telephone (8.3%, 1/12), or email (8.3%, 1/12). The most commonly reported problem was related to notifications (66.7%, 8/12).

## 4. Discussion

This study sought to evaluate the feasibility of digital health technology in supporting children (aged 10 to 18 years) living with JIA. The intervention was designed to enable real-time remote monitoring of pain levels, medication adherence, and physical activity levels by the participant, parent/carer, and the PR research team. The data attained by the participants wearing a smartwatch and using the customised mobile app was captured by the web-based platform and presented as an interface of daily and monthly graphs. Thus, it is suggested that collecting real-time data from children is achievable, despite the many challenges exposed in this study.

### 4.1. App Usage

Interestingly, similar rates of app usage were identified for the pain level module (68%) and the physical activity module (69.7%). Ultimately, the data captured on the web-based platform were reliant on the participants’ engagement with the intervention [38], yet there were differences in how the data were collected. The pain level data needed manual input, while the physical activity data were generated automatically. However, it is not clear whether this rate of app usage achieved a fair or good response due to the paucity of research benchmarking acceptable usage rates [38,39]. Although qualitative research does suggest that even brief periods of engagement with a health intervention can be beneficial to the user, users still gain knowledge or learn strategies that could be practiced without additional support from the intervention [40].

In contrast, app usage for the medication module was noticeably lower (20.7%). Participants reported several issues with the system, hindering app usage. Unlike the pain module, participants could not enter their responses earlier or later than their pre-selected administration times because the system aimed for medications to be taken at the right time. This may be achievable in a clinical setting but may not always be realistic in a real-world setting. Participants found this restrictive timeframe inconvenient, especially if they were out on the weekend. Participants wanted the system to be adaptable to their events, allowing their responses to be pre-entered or backdated when needed. Instead, the medication module became confusing. Participant 3 explains, “Sometimes the medication screen would not appear when I opened the app”. This was because the app had become inactive. This resulted in three participants failing to use the medication module properly and another three participants requesting to change their administration times. However, once the preselected times were entered into the system, they could not be changed (16.7%, 2/12) or removed (8.3%, 1/12). Exploring these reasons for non-app usage is important because different target groups have different needs [38], emphasising the importance of a user-led design to improve app usage. This understanding resulted in system improvements, allowing for flexible and changeable administration times.

### 4.2. Confirmation of Data Integrity

Remotely monitoring patients from home requires a data communication system between the patient and the healthcare team. Therefore, it is essential to establish that there are no errors due to data transmission. For this study, InteractiveClinics data communication between the watch, app, and web-based platform for the pain and medication module was error-free. This finding is important because most apps to date have been designed by software developers, not health professionals or academics [41], and they have not provided the multimodal approach needed to support and monitor chronic disease. For example, combining medication administration and symptom monitoring [42], as very few systems can collate the large amount of data collected from smartwatches and smartphones [43]. Instead, to compensate, most digital health researchers have needed to use multinational manufacturer sites [43] and develop their interventions towards either Apple or Android devices, not both [44]. For many, this has impended more complex app development and raised ethical concerns around participant consent, privacy, data storage, and security [45], especially for children.

However, improvements were also needed because the InteractiveClinics data communication system for the physical activity module was not error-free. There was a 10% reporting error, resulting in the underestimation of physical activity levels or missing data on the web-based platform. Also, half of the participants (50%, 6/12) reported not being able to view their physical activity levels in real time directly from the app. InteractiveClinics was meant to run as a background app, where the contents are automatically refreshed. These problems were exposed because InteractiveClinics was a third-party app and did not directly collect raw data from the watch via Bluetooth. Instead, it was reliant on Wi-Fi or cellular data to support communication and the transference of data from the Apple Health Kit. This enabled the synchronisation of the data in the InteractiveClinics app and web-based platform, where the programming interface created a reactive response in the app. Understandably, there is a growing concern among many third-party developers to improve this interoperability by standardising the exchange of data [46]. This communication pathway can also be easily impaired by breaks in internet connectivity or device fragmentation, inhibiting transfers [46]. Participants in this study were only supplied with cellular data on the phone rather than over Wi-Fi, which can become unreliable indoors [47]. It was also observed, when all the watches and phones were returned after the study period, that not all devices had received their latest manufacturer updates whilst out on loan. To compensate and support data transfer during the study, the physical activity module needed to be regularly opened by the participants. Certainly, this is an area that requires further technological advancement in this emerging health field.

### 4.3. Preliminary Interpretation

#### 4.3.1. Pain Level Module

Across the study period, the web-based platform captured the participants’ pain scores. On average, mild pain scores (mean: 2.9 out of 10, SD: 1.8, range: 0.3 to 6.2 out of 10) were reported. This ongoing monitoring of pain is important because pain is the most common and distressing symptom of JIA [15].

#### 4.3.2. Medication Adherence

Across the study, low rates of medication adherence were depicted daily (39%) and weekly (37.5%). However, there were a number of issues identified in this study that negatively affected accurate reporting rates. This resulted in three participants (25%, 3/12) failing to use the medication module properly and another three (25%, 3/12) needing to re-connect the app to the web-based platform. Although this only took a few seconds to rectify, it was completed remotely by sending a new access code to the participant by text message to re-enter into the medication module. Participants were unable to backdate their responses, resulting in missing administration responses.

#### 4.3.3. Physical Activity Module

For the physical activity module, preliminary results illustrated low levels of moderate-to-vigorous levels of physical activity (mean: 11.8, SD: 13.5 min per day). These results are similar to other JIA studies, demonstrating levels far below the recommended 60 min per day [48], reinforcing the importance of improving physical activity levels for children with JIA.

The results from the physical activity module are also encouraging, because most research to date in paediatrics has focused on wearable devices such as pedometers or activity trackers rather than smartwatches [49,50,51]. Therefore, these findings will add to the growing body of literature supporting the potential of the biometers within the smartwatch to monitor healthcare in a home setting [21].

### 4.4. Watch Wearing Behaviour

Across the study period, watch wearing behaviour ranged from 28.6% to 100% of the expected days. To date, there has been limited research reporting on watch wearing behaviour; instead, research has focused mainly on usability [52,53]. Understanding the mechanisms that drive continuous use is important [53], as differences have been exposed between adults and children. For example, a study of college students (aged 18–37 years) identified high rates of watch wearing behaviour (89.8%) across their study period and different usage patterns on weekdays (11.32 h, SD: 3.53) and weekends (8.66 h, SD: 3.60) [52]. While a study on children (aged 10–15 years) with neurodevelopmental disorders reported lower watch wearing patterns (5.66 h per day, SD: 3) because children only wanted to wear the watch while completing their school day [54]. Interestingly, in this study, the three oldest female participants had the lowest rate of watch wearing behaviour (28.6% to 35.7%) because they disliked the feeling of the watch on their wrist, and it is yet unclear whether this was due to JIA-related pain sensations. While the two youngest participants had the highest rate of adherence (100%), some studies do suggest that higher rates of app usage are linked to younger participants and digital literacy [38], and lower rates are due to uncomfortability, aesthetic concerns, and daily routines [52]. Indeed, this is an area in need of further research to fully understand these reasons.

### 4.5. Technical Issues

An essential ingredient in supporting this feasibility study was allowing the participants access to ongoing technical support. This enabled most barriers to be promptly and smoothly addressed as they arose. For example, five participants (41.7%, 5/12) became confused when confirming pain entries and needed additional training. How participants sought this technical support differed from previous JIA studies. The preferred method of contact was text messaging instead of phone calls or emails [55]. It is not a surprising change, considering the recent rise in smartphone ownership, making text messages the most used form of communication by adolescents [56]. However, recommending the use of text messages in clinical practice is beyond the scope of this research. It would require additional precautions for this age group (aged 10–18 years) to ensure the required safety and ethical standards are met and policy guidelines are incorporated [57], in particular whilst attending school.

In addition, the capability of InteractiveClinics, allowing customised notifications to be sent at a time nominated by participants, un-intentionally, provided the research team with an exact time a participant could be reached to provide technical support, removing any impact on school or extra-curricular activities and allowing their problems hindering adoption to be immediately fixed. For example, when the research team noticed pain levels not recorded on the web-based platform, in fact, a recent systematic review highlighted the importance of human contact for eHealth and mHealth interventions to promote engagement [55].

### 4.6. Limitations

#### 4.6.1. Internal Limitations

There are several limitations to this study. First, this study used convenience sampling to gain quick feedback on the feasibility of InteractiveClinics to support development. This sampling method limits generalisability.

Second, participants enrolled in this study understood that they needed to use and provide their opinions on functionality. For many participants, problems began emerging immediately. This resulted in several participants providing feedback within the first few days of the study period; therefore, participants may have lost interest, impacting adoption of the intervention.

Conversely, gaining early feedback helped others with app usage. For example, five participants (41.7%, 5/12) were confused when confirming their pain entries. They identified that after entering their pain level on the *eVAS*, there was no endpoint to the pain assessment. The *eVAS* response produces a numerical number (between 0 and 10) on the same screen, with no confirmation button. Instead, a ‘continue’ button appeared, taking the participants to another screen to take another sample. To overcome this problem, participants were instructed to ignore the ‘continue’ button and were assured their pain level had been registered on the web-based platform. This fault was then highlighted to all newly recruited participants in their training session. This also led to future improvements in the design of the pain modules development, because a clear confirmation button will be provided and a visual confirmation response will appear in the testing stage.

#### 4.6.2. External Limitations

First, all participants were asked during training to link the phone provided to their home Wi-Fi to improve connectivity and ensure that they had enough cellular data to participate in the study when they were out. Unfortunately, two participants (16.7%, 2/12) had used all of the phone’s cellular data (3 Gigabytes) by day 4 of the 2-week study period and day 14 of the 4-week study period. This impaired their ability to engage with the intervention because no additional credit was accessible for the participants. When these phones were returned, it was revealed that these data had been used unnecessarily and excessively in the weekly screen time summary (>8 h per week of personal streaming). Concerningly, if digital health aims to reduce the long-standing inequalities that exist in gaining access to healthcare, the availability of Wi-Fi or cellular data to connect to the internet needs to be considered [58]. Fortunately, the price of data is significantly reducing, and many countries such as Bolivia, Libya, and South Africa are, for the first time, reaching the United Nations’ target of affordable data [59].

Second, eight participants (66.7%, 8/12) reported ongoing notification errors that limited their app usage. Participant 1 explains, “For the majority of days, I did not received notifications, and it was hard to respond”. Notification delivery and responses were reliant on stable internet connectivity to promote communication between the watch, app, and web-based platform. If participants did not link the phone to their home Wi-Fi and used only the phone’s cellular data, this connectivity can be impaired whilst indoors. Even despite the recent advancements in the 5G network, Wi-Fi connectivity remains superior within the home [47].

Notification problems also occurred due to device barriers. Some participants did not feel the vibration on their arm triggered by the smartwatch when a new notification arrived. Notifications were also time-sensitive, so if they were missed, “messages disappear” (Participant 10) from the home screen of the smartwatch due to the model of the smartphone used in this study. For the last four participants enrolled in this study, this barrier was overcome by instructing them to swipe down on their watch or phone to re-review past notifications. Participants also needed to answer each notification separately and directly on the phone. This approach increased the medication reporting rate for these participants to 72.3% (34/44) against the expected number of entries. If notifications were correctly delivered, they pragmatically reminded users to engage with the intervention [60], increasing usage [61], and therefore decreasing the risk of drop-out.

By identifying the challenges participants faced, an opportunity was provided to promptly fine-tune InteractiveClinics with the aim of increasing engagement because the success of a digital health intervention is dependent on usage in order to meet the expected end goal of improving treatment adherence and JIA-related symptom monitoring.

### 4.7. Clinical Importance

An important clinical finding in this study was that emerging digital technology has the ability to monitor PRN medications remotely through a web-based platform. Participants recording additional medications highlighted the participants immediate needs [62]. Typically, PRN medications are used for symptom control rather than providing treatment for disease. Therefore, capturing these real-time data records important moments that may have been missed during the healthcare consultation. Instead, by acknowledging PRN medications, the appropriate follow-up can be provided, as an alternative intervention may be needed [63].

### 4.8. Future Direction

Although this study only conducted a preliminary analysis of the data collected on the web-based platform, the potential health data that can be gathered from children living with a chronic health condition were demonstrated. Further research is now needed to ensure the suggested changes made to InteractiveClinics improve the users experience, create a user-led design, and increase app usage to ensure the intervention achieves what was indented.

Importantly, this study also identified several major challenges that digital healthcare faces, stemming from the external digital eco-system. For example, poor internet connectivity impaired notification delivery and the physical activity module’s data integrity. In fact, a recent scoping review identified poor internet connectivity as a common problem in many digital health studies [64]. Therefore, to ensure the collection of medical-grade data that can be used in clinical practice, a concerted effort is needed by all stakeholders to remove any external barriers [65]. Especially considering the recent expanse of third-party developers and the wide range of smartwatches now available globally that will require standardised interoperability [46].

## 5. Conclusions

The feasibility of using a web-based platform to support integrated care between a child, parent, and healthcare team was demonstrated by remotely monitoring real-time data through emerging digital health technologies. In this study, using a smartwatch and customised mobile app, daily pain levels, medication adherence, PRN medication needs, and physical activity were remotely monitored on a web-based health platform. This multimodal intervention is an important step towards supporting the management of JIA through digital health. However, further research is still needed to improve the user experience, create a user-led design, and increase adoption by children.

## Figures and Tables

**Table 2:** Schedule of data collection.

	Daily on the Web-Based Platform	When Support Required
Pain		
eVas [32,33,34]	✓ ^a^	
Medication adherence		
Medication administration	✓	
Reasons for non-adherence	✓	
PRN medication administration	✓	
Physical activity levels		
Kilojoules—light physical activity levels	✓	
Moderate-to-vigorous physical activity levels	✓	
Hourly stand goals	✓	
Watch wearing behaviour	✓	
Technical errors		✓

a ✓: confirmining data collected.

## Data Availability

The data presented in this study are available on request from the corresponding author. The data are not publicly available due to the research being conducted on children.
